# *SYTL3*–*SLC22A3* Single-Nucleotide Polymorphisms and Gene–Gene/Environment Interactions on the Risk of Hyperlipidemia

**DOI:** 10.3389/fgene.2021.679027

**Published:** 2021-07-21

**Authors:** Peng-Fei Zheng, Rui-Xing Yin, Xiao-Li Cao, Yao-Zong Guan, Guo-Xiong Deng, Bi-Liu Wei, Chun-Xiao Liu

**Affiliations:** ^1^Department of Cardiology, Institute of Cardiovascular Diseases, The First Affiliated Hospital, Guangxi Medical University, Nanning, China; ^2^Guangxi Key Laboratory Base of Precision Medicine in Cardio-Cerebrovascular Disease Control and Prevention, Nanning, China; ^3^Guangxi Clinical Research Center for Cardio-Cerebrovascular Diseases, Nanning, China; ^4^Department of Neurology, The First Affiliated Hospital, Guangxi Medical University, Nanning, China

**Keywords:** haplotype, synaptotagmin like 3, environmental factor, single nucleotide polymorphism, solute carrier family 22 member 3, hyperlipidaemia, interaction

## Abstract

The current study aims to further delineate the associations between the synaptotagmin-like 3 (*SYTL3*) and solute carrier family 22 member 3 (*SLC22A3*) single-nucleotide polymorphisms (SNPs) and their haplotypes and gene–gene (G × G)/environment (G × E) interactions on the risk of hyperlipidemia (HLP) in the Maonan and Han ethnic groups. Genotype distribution among the *SYTL3–SLC22A3* SNPs in 2,829 individual patients bearing no relationship to each other (Han, 1,436; Maonan, 1,393) was analyzed utilizing next-generation sequencing techniques. The genotype frequencies of the rs6455600, rs2129209, and rs446809 SNPs were varied between the two ethnic groups (*P* < 0.05–0.001). Various SNPs were correlated with serum levels of triglyceride (TG; rs446809), total cholesterol (TC; rs6455600, rs2129209, and rs539298), and low-density lipoprotein cholesterol (LDL-C; rs446809) among the Han population, whereas various SNPs were also correlated with TC (rs6455600 and rs539298), TG (rs446809), and LDL-C (rs446809) levels in the Maonan ethnic group (*P* < 0.008–0.001). One part of haplotypes resulted in worsened HLP-related morbidity in the Han (*SYTL3* A-C-A-A; *SLC22A3* A-A and A-G; and *SYTL3–SLC22A3* A-C-A-A-A-A and A-C-A-A-A-G) and Maonan (*SYTL3* A-C-A-A; *SLC22A3* A-A and A-G; and *SYTL3–SLC22A3* A-C-A-A-A-A, G-T-C-A-A-A, and G-T-C-A-C-A) ethnic groups, whereas another part of haplotypes lowered HLP-related health risks in the Han (*SLC22A3* C-A and C-G and *SYTL3–SLC22A3* A-C-A-A-C-A, A-C-A-A-C-G, and G-T-C-A-C-A) and Maonan (*SLC22A3* C-G and *SYTL3–SLC22A3* A-C-A-A-C-G) ethnic groups. We discovered that the *SYTL3–SLC22A3* SNPs and their haplotypes were associated with serum lipid levels and the risk of HLP in our studied populations.

## Introduction

Coronary artery disease (CAD) has become a prominent cause of morbidity, mortality, disability, high healthcare costs, and functional deterioration and accounts for approximately 30% of all deaths worldwide (Yokokawa et al., 2011; Finegold et al., 2013; Houston, 2018). Hyperlipidemia (HLP) is a major risk factor for CAD and its complications. Comprehensive lipid-lowering therapy is recommended for patients with CAD by the 2013 American College of Cardiology (ACC)/American Heart Association (AHA) guidelines for the treatment of blood cholesterol to reduce the risk of cardiovascular events (Ray et al., 2014). The guidelines emphasize that lipid-lowering therapy should not focus solely on decreasing low-density lipoprotein cholesterol (LDL-C) levels. Several compelling studies proved that lowering total cholesterol (TC) (Chapman et al., 2011), triglyceride (TG) (Chapman et al., 2011), and LDL-C (Can et al., 2010) levels is more effective in reducing cardiovascular risk than lowering LDL-C levels alone (Ferrieres et al., 2013). HLP appears to be strongly dependent on a person’s genotype, with 40–60% of serum lipid profile variations attributed to hereditary causes (Heller et al., 1993). Hence, discovering novel lipid-related genes is an important step toward developing novel lipid-lowering drugs that may offer a more personalized approach in combating HLP.

Currently, several compelling genes closely related to serum lipid parameters including the synaptotagmin-like 3 (*SYTL3*) and solute carrier family 22 member 3 gene (*SLC22A3*) have been characterized in the European population by genome-wide association studies (GWASes) (Ober et al., 2009). *SYTL3* (also named as *SLP3*, HGNC:15587, OMIM: 608441, gene ID: 94120) is encoded in chromosome 6q25.3 (exon count: 22), and its resultant protein plays a crucial role in vesicular trafficking. Several studies have pointed that the transport of proteins and lipids between eukaryotic cells via endocytosis and secretion is mainly facilitated by vesicular trafficking (Agmon and Stockwell, 2017; Stefan et al., 2017). Muse et al. (2017) documented that *SYTL3* was correlated with acute myocardial infarction (AMI); however, the underlying mechanism is unclear. *SLC22A3* (also known as *EMT*, *EMTH*, and *OCT3*; OMIM: 604842; gene ID: 6581; HGNC:10967) is a gene found on chromosome 6q25.3 (exon count: 15) and functions to produce the organic cation transporter 3 that belongs to a family of peripheral membrane proteins and is critical in biogenic histamine synthesis and deactivation (Verhaagh et al., 1999; Schneider et al., 2005). As a potent pro-inflammatory mediator, histamine enhances LDL-C deposition in vascular endothelial cells by inducing cytokines, adhesion molecules, chemokines, and other inflammatory mediators. Coupled with the increased permeability of these cells, histamine produces a conductive environment for atherosclerotic plaque formation (Kimura et al., 2004; Rozenberg et al., 2010). Functional studies have shown that SLC22A3 silencing could significantly inhibit histamine synthesis, synthesis of pro-inflammatory mediators (MCP-1, IL-8, and IL-6), and mononuclear cell infiltration and may also impair leukocyte–endothelial interaction (Li et al., 2015). *SLC22A3* is more widely distributed and is found in the skeletal muscle, heart, brain, and placenta. *SLC22A3* is highly expressed in the human heart, with the strongest *SLC22A3* immunoreactivity found in vascular endothelial cells (Chen et al., 2010; Solbach et al., 2011). A number of studies have showed that the *SLC22A3* rs2048327, rs1810126, and rs3088442 single-nucleotide polymorphisms (SNPs) contributed toward depressed CAD risk through downregulation of *SLC22A3* transcription and protein levels (Li et al., 2015; Zhao et al., 2017). Chen et al. (2019) found that *SLC22A3* could serve as a mediator for the genetic correlation between lipid metabolism and CAD. Nevertheless, the correlation between serum lipid profiles, *SYTL3–SLC22A3* SNPs, and ethnicity has yet to be completely documented.

China is a country comprising of multiple ethnicities including the predominant Han group along with 55 other ethnicities. Of these minorities, the Maonan represents the 37th largest, with a total population of 107,166, as recorded in the Sixth National Population Census (2010). Most people of this ethnic group live in the Huanjiang Maonan Autonomous County of Guangxi Zhuang Autonomous Region. The Maonan people vary widely with the local Han population in terms of dietary and lifestyle habits. They are an ideal cohort for genetic variation research because they rarely intermarry with other ethnic groups (Li et al., 2009). Although the association between blood lipid profiles and several genotypes of the *SYTL3–SLC22A3* cluster has been documented in the European population, the correlation of the new alternates and their haplotypes with the possibility of HLP in the Han and Maonan ethnic groups has never been reported previously. Thus, this study aims to (i) explore the connection of the *SYTL3* (rs9364496, rs6455600, rs2129209, and rs9456350) and *SLC22A3* (rs446809 and rs539298) SNPs and serum lipid profiles between the Maonan and Han ethnicities; (ii) assess the correlation of their haplotypes with HLP morbidity between both ethnic groups; (iii) detect the potential gene–gene (G × G) and gene–environment (G × E) interactions that may impact the clinical HLP manifestations in the two ethnic groups.

## Materials and Methods

### Subjects

A total of 1,436 unrelated people (668 males, 46.52% and 768 females, 53.48%; 749 normal, 52.16% and 687 HLP, 47.84%) of the Han ethnic group and 1,393 unrelated individuals (624 males, 44.80% and 769 females, 55.20%; 631 normal, 45.30% and 762 HLP, 54.70%) of the Maonan ethnic group were randomly selected from our previously stratified randomized specimen bank (Wang et al., 2016; Bin et al., 2017). All participants were occupants of the same location in the Guangxi Zhuang Autonomous Region, which is the Huanjiang Maonan Autonomous County of China. All individuals were farmers, ages from 20 to 88. Both Han and Maonan ethnic groups possessed similar sex ratio, age distribution, and average age (55.38 ± 12.46 vs. 56.35 ± 15.34; respectively). All subjects had no previous history of type-2 diabetes mellitus (T2DM), ischemic stroke, CAD, and myocardial infarction. None of the subjects was taking any medication that impacted blood lipid profiles. All protocols were vetted by the Ethics Committee of the First Affiliated Hospital, Guangxi Medical University (No. Lunshen-2014 KY-Guoji-001, March 07, 2014). All participants provided signed informed consent prior to the study.

### Epidemiological Analysis

Epidemiological investigation was performed in compliance to protocols previously established (Tao et al., 1992). Questionnaires allowed for documentation of relevant demographic and lifestyle information. Three separate subgroups each of smoking status [0 (non-smoker), <20 cigarettes/day, and ≥20 cigarettes/day] and alcohol consumption [0 (non-drinker), <25 g/day, and ≥25 g/day] were formed. The waist circumference, height, blood pressure, and body mass index (BMI) were assessed based on previous research (Guo et al., 2015a).

### Biochemical Assays

A fasting venous blood sample of 5 ml was collected from each participant. A portion of the sample (2 ml) was collected in a test tube and used to measure serum lipid levels. The rest of the sample (3 ml) was collected into a test tube containing anticoagulants (13.20 g/l tri-sodium citrate, 14.70 g/l glucose, and 4.80 g/l citric acid) and utilized to extract deoxyribonucleic acid (DNA). Methods for measuring serum LDL-C, apolipoprotein A1 (ApoA1), apolipoprotein B (ApoB), TC, TG, and HDL-C were described in detail in a previous study (Sun et al., 2015). All measurements were performed using an autoanalyzer (Type 7170A; Hitachi Ltd., Tokyo, Japan) in the Clinical Science Experiment Center of the First Affiliated Hospital, Guangxi Medical University (Guo et al., 2015b).

### SNP Selection

Six SNPs located on the *SYTL3* and *SLC22A3* were chosen using a number of pre-determined criteria: (1) *SYTL3–SLC22A3* cluster was selected from previous GWASes associated with blood lipid levels. (2) Tagging SNPs were identified via Haploview (Broad Institute of MIT and Harvard, Cambridge, MA, United States, version 4.2), and potential lipid metabolism-associated functional SNPs were predicted using the latest version of the 1000 Genomes Project database. (3) More completely, details of the selected SNPs were collected from the NCBI dbSNP Build 132. (4) Regarding the SNP selection, we also referenced to a previous study by Ober et al. (2009), and the minor allele frequency (MAF) of all SNPs was more than 1% and associated with blood lipid profiles in previous study. (5) Six SNPs of *SYTL3* rs9364496, rs6455600, rs2129209, and rs9456350 and *SLC22A3* rs446809 and rs539298 were selected using the block-based method, which involves marking the association of linkage disequilibrium (LD) among chosen SNPs (*r*^2^ > 0.8).

### DNA Amplification and Genotyping

White blood cells were used for genomic DNA extraction with phenol–chloroform (Yin et al., 2011). All obtained DNA samples were numbered and maintained at −20°C until further studies were carried out. Next-generation sequencing technology (NGS) was used to analyze the genotypes of the six selected SNPs at the Center for Human Genetics Research, Shanghai Genesky Bio-Tech Co. Ltd., Shanghai, China (Onda et al., 2018). Supplementary Table 1 depicts all relevant primer sequences.

### Diagnostic Criteria

The following normal values were implemented in our research: serum ApoA1 (1.20–1.60 g/l), TG (0.56–1.70 mmol/l), LDL-C (2.70–3.10 mmol/l), TC (3.10–5.17 mmol/l), HDL-C (1.16–1.42 mmol/l), ApoA1/ApoB ratio (1.00–2.50), and ApoB (0.80–1.05 g/l). HLP was diagnosed when TC levels were more than 5.17 mmol/l and/or TG levels were more than 1.70 mmol/l (Guo et al., 2016). Diabetes (Alberti and Zimmet, 1998), body weights (obese, normal, or overweight) (Zhou, 2002), and hypertension (Chalmers et al., 1999; Whitworth, 2003) were diagnosed based on previously established criteria.

### Statistical Analyses

The SPSS (version 22.0, IBM Corp., Armonk, NY, United States) was used for all data analyses. The information of measurement parameters is depicted in terms of mean ± SD with the exception of TG levels, which is depicted in terms of median and interquartile ranges. Intergenotype variances between the six SNPs as well as between the proportion of alcohol consumers and smokers were evaluated using the chi-square test. Several common characteristics were tested by independent-sample *t-*test or Kruskal–Wallis test between both ethnicities. Haploview (Broad Institute of MIT and Harvard, Cambridge, MA, United States; version 4.2) allowed for assessment of the pairwise LD (measured by *r*^2^), Hardy–Weinberg equilibrium (HWE), and the frequencies of haplotypes or gene–gene interaction haplotypes in two ethnic groups. HLP diagnosis and its correlation to genotypes/haplotypes were assessed using unconditional logistic regression analysis. Analysis of covariance (ANCOVA) was used to assess the association between genotypes/haplotypes and serum lipid profiles, and *P* < 0.008 or *P* < 0.0045 (equivalent to *P* < 0.05 after adjusting for six SNPs or 11 haplotypes in independent tests by Bonferroni correction) was determined to be possessing significant statistical significance. Related parameters including blood pressure, smoking, blood glucose, sex, BMI, age, and alcohol consumption as covariables were adjusted for the correlation analysis. The generalized multifactor dimensionality reduction (GMDR) was used to determine the ideal combination among gene–gene/environment exposures, haplotype–haplotype/environment exposures, and SNP–SNP/environment exposures (Lou, 2015). GMDR reduces high-dimensional genetic data to a single dimension by exploring interaction models through cross-validation and using maximum likelihood estimates to calculate the score-based statistics of each participant. A good model would ideally possess a cross-validation constancy (CVC) value close to 10, which allows for identification of the best model among all likely candidates. Balanced accuracy testing scores between 0.50 (model prediction results are no better compared to chance) and 1.00 (perfect model prediction results) are indicative of the extent of the case-control status. Permutated *P*-values for these models were obtained by performing 1,000 permutations. The GMDR analyses included all haplotypes, SNPs, and numerous environmental modifiers, such as smoking, drinking, hypertension, and BMI. The analyses were further adjusted for study population, gender, and age. Statistical significance was ascertained with a *P*-value of <0.05. Multivariable linear regression analyses implementing stepwise modeling were carried out to discern associations between genotypes, alleles, and various modifiable environmental components with blood lipid profile in the Han and Maonan ethnic groups. Likewise, statistical significance was deemed to be achieved if *P* < 0.05. An interactive heat map with various serum lipid profile parameters was constructed using the R software (version 3.3.0) (Zhao et al., 2014).

## Results

### Common and Biochemical Characteristics in the Han and Maonan Ethnic Groups

As shown in Table 1, there were several differences in serum lipid levels and general parameters between the Han and Maonan ethnic groups. We noticed that the Maonan population possessed higher levels of LDL-C, diastolic blood pressure, ApoB, systolic blood pressure, TC, TG, weight, alcohol consumption, pulse pressure, and BMI in contrast to the Han group (*P* < 0.05–0.001). However, serum ApoA1, HDL-C levels, and the ApoA1/ApoB ratio were decreased in the Maonan ethnicity than those in the Han ethnic group (*P* < 0.05–0.001). Gender ratio, age distribution, height, glucose, the proportion of smokers, and waist circumference were similarly distributed between both ethnic groups.

**TABLE 1:** Comparison of demographic and lifestyle characteristics and serum lipid levels between the Han and Maonan populations.

Parameter	Han			Maonan			*P*_Han vs. Maonan_	*P*_Han_	*P*_Maonan_
		
	All	Normal	HLP	All	Normal	HLP			
Number	1,436	749	687	1,393	631	762			
Male/female^3^	668/768	337/412	331/356	624/769	273/358	351/411	0.358	0.226	0.296
Age (years)^1^	56.61 ± 12.23	56.42 ± 12.67	56.81 ± 11.73	55.98 ± 14.67	54.54 ± 15.64	56.36 ± 13.81	0.220	0.549	0.300
Height (cm)^1^	152.09 ± 7.71	152.37 ± 7.46	151.79 ± 7.96	151.58 ± 7.31	151.35 ± 7.37	151.78 ± 7.25	0.074	0.159	0.267
Weight (kg)^1^	52.50 ± 9.30	51.99 ± 9.13	52.80 ± 10.29	53.68 ± 10.74	52.08 ± 10.35	55.00 ± 10.89	0.002	0.003	4.00E-7
Body mass index (kg/m^2^)^1^	22.66 ± 3.60	22.39 ± 3.594	22.96 ± 3.59	23.28 ± 4.03	22.68 ± 4.12	23.78 ± 3.90	1.74E-5	0.003	4.18E-7
Waist circumference^1^	75.49 ± 8.23	74.89 ± 8.27	76.15 ± 8.15	75.62 ± 9.73	73.10 ± 9.28	77.70 ± 9.62	0.719	0.004	5.61E-19
Smoking status [*n* (%)]^3^									
Non-smoker	714 (49.72)	511 (68.22)	203 (29.55)	729 (52.33)	369 (58.48)	360 (47.24)			
≤20 cigarettes/day	317 (22.08)	105 (14.02)	212 (30.86)	323 (23.19)	123 (19.49)	200 (26.25)			
> 20 cigarettes/day	405 (28.20)	133 (17.76)	272 (39.59)	341 (24.48)	139 (22.03)	202 (26.51)	0.080	2.77E-47	1.27E-4
Alcohol consumption [*n* (%)]^3^									
Non-drinker	860 (59.89)	504 (67.29)	356 (51.82)	691 (49.61)	331 (52.46)	360 (47.24)			
≤25 g/day	353 (24.58)	172 (22.96)	181 (26.35)	494 (35.46)	236 (37.40)	258 (33.86)			
>25 g/day	223 (15.53)	73 (9.75)	150 (21.83)	208 (14.93)	64 (10.14)	144 (18.90)	1.61E-11	1.61E-11	3.00E-5
Systolic blood pressure (mmHg)^1^	128.18 ± 19.66	127.11 ± 19.57	130.98 ± 23.67	129.35 ± 19.70	127.50 ± 23.23	133.86 ± 23.66	0.001	0.031	5.21E-7
Diastolic blood pressure (mmHg)^1^	79.56 ± 11.11	78.48 ± 11.29	80.73 ± 10.80	81.13 ± 12.41	79.96 ± 12.57	82.10 ± 12.20	3.75E-4	1.21E-4	0.001
Pulse pressure (mmHg)^1^	48.63 ± 14.89	47.71 ± 14.65	48.62 ± 15.14	49.85 ± 17.10	47.53 ± 16.59	51.77 ± 17.29	0.002	0.011	3.98E-6
Glucose (mmol/l)^1^	6.09 ± 1.67	6.02 ± 1.50	6.16 ± 1.83	6.11 ± 1.31	6.04 ± 1.12	6.16 ± 1.44	0.688	0.109	0.092
Total cholesterol (mmol/l)^1^	4.66 ± 1.04	4.16 ± 0.76	5.20 ± 1.02	4.96 ± 1.07	4.35 ± 0.53	5.47 ± 1.13	2.61E-14	1.30E-91	7.60E-98
Triglyceride (mmol/l)^2^	1.10 (0.80)	1.00 (0.50)	1.45 (1.37)	1.15 (0.95)	0.90 (0.5)	1.64 (1.27)	0.004	4.63E-31	9.11E-66
HDL-C (mmol/l)^1^	1.71 ± 0.49	1.75 ± 0.0.56	1.63 ± 0.0.41	1.58 ± 0.43	1.64 ± 0.42	1.53 ± 0.44	1.90E-13	0.017	0.032
LDL-C (mmol/l)^1^	2.80 ± 0.84	2.65 ± 0.75	3.07 ± 0.89	2.95 ± 0.86	2.43 ± 0.49	3.11 ± 0.97	6.75E-5	2.85E-22	4.84E-54
ApoA1 (g/l)^1^	1.38 ± 0.25	1.39 ± 0.26	1.34 ± 0.25	1.33 ± 0.23	1.36 ± 0.22	1.30 ± 0.25	1.82E-7	3.94E-4	2.14E-8
ApoB (g/l)^1^	0.85 ± 0.21	0.81 ± 0.21	0.88 ± 0.21	0.88 ± 0.24	0.77 ± 0.16	0.98 ± 0.25	1.42E-4	8.73E-10	7.95E-67
ApoA1/ApoB^1^	1.73 ± 0.64	1.83 ± 0.70	1.62 ± 0.54	1.62 ± 0.53	1.81 ± 0.44	1.46 ± 0.54	4.30E-7	3.85E-10	4.10E-36

**HDL-C*, high-density lipoprotein cholesterol; *LDL-C*, low-density lipoprotein cholesterol; *Apo*, apolipoprotein; *HLP*, hyperlipidemia. The value of triglyceride is presented as median (interquartile range) for non-normal distribution.*

*^1^Mean ± SD determined by *t*-test.*

*^2^Median (interquartile range) tested by the Wilcoxon–Mann–Whitney test.*

*^3^The rate or constituent ratio between the different groups was analyzed by the chi-square test.*

### Common and Biochemical Characteristics Between Normal and HLP Populations in Both Ethnic Groups

As shown in Table 1, further subgroup analyses found that there were significant differences in blood lipid levels and general parameters between the normal and hyperlipidemia populations in both ethnic groups. We noticed that weight, systolic blood pressure, TC, waist circumference, ApoB, the proportion of smokers, BMI, alcohol consumption, TG, diastolic blood pressure, pulse pressure, and LDL-C were raised in patients with HLP in contrast to healthy participants in both ethnic groups. Conversely, patients with HLP had lower levels of ApoA1, HDL-C, and ApoA1/ApoB ratio in contrast to healthy individuals; these findings were similar irrespective of ethnic groups. Interestingly, there was no any stark difference in height, gender, age distribution and glucose levels between HLP patients and normal subjects in both the Han and Maonan ethnic groups.

### Genotypic and Allelic Frequencies of Six SNPs Between the Normal and HLP Populations in Both Ethnic Groups

**FIGURE 1:**
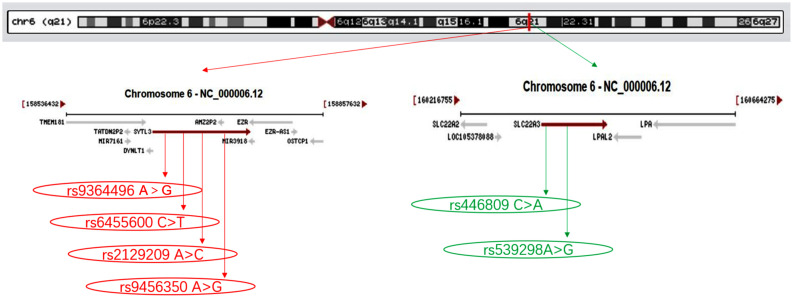
The positions of the synaptotagmin-like 3 (*SYTL3*) and solute carrier family 22 member 3 (*SLC22A3*) mutations.

Figure 1 depicts the location of six detected SNPs of *SYTL3–SLC22A3* cluster in a closely genomic area of chromosome 6. All six SNPs were distributed in a manner complying with HWE across both the Maonan and Han groups (*P* > 0.05 for all). As shown in Table 2, several SNPs possess allelic and/or genotypic frequencies that were significantly varied between the two ethnic groups (rs6455600, rs2129209, and rs446809); between the normal and HLP populations in the Han (rs6455600, rs2129209, rs446809, and rs539298) and Maonan groups (rs6455600, rs446809, and rs539298; *P* < 0.05–0.001, respectively). These differences may partly account for the differences in blood lipid levels and the risk of hyperlipidemia between the Han and Maonan populations.

**TABLE 2:** The association between the *SYTL3* and *SLC22A3* polymorphisms and hyperlipidemia in the Han and Maonan groups.

SNP	Genotype	Han			Maonan			*P_Han vs. Maonan_	*P_Han_	OR (95% CI)	**P_Han_	*P_Maoan_	OR (95% CI)	**P_Maonan_
		All (*n* = 1,436)	Normal (*n* = 749)	HLP (*n* = 687)	All (*n* = 1,393)	Normal (*n* = 660)	HLP (*n* = 733)							
*SYTL3*	A/A	876 (60.9%)	463 (61.8%)	413 (60.1%)	868 (62.3%)	419 (63.5%)	449 (61.3%)							
rs9364496 G > A	G/A + G/G	560 (39.1%)	286 (38.2%)	274 (39.9%)	525 (37.7%)	241 (36.5%)	284 (38.7%)	0.47	0.51	1.12 (0.89–1.42)	0.34	0.39	1.00 (0.78–1.29)	0.97
	MAF	635 (22.1%)	332 (22.2%)	303 (22.1%)	601 (0.22)	277 (0.21)	324 (0.22)	0.62	0.94			0.48		
	*P*_HWE_	0.44	0.06	0.38	0.08	0.10	0.39							
*SYTL3*	C/C	708 (49.3%)	333 (44.5%)	375 (54.6%)	854 (61.3%)	369 (55.9%)	306 (41.8%)							
rs6455600 C > T	C/T + T/T	728 (50.7%)	416 (55.5%)	312 (45.4%)	539 (38.7%)	291 (44.1%)	427 (58.2%)	1.38E-10	1.26E-4	0.70 (0.55–0.88)	0.002	1.28E-7	1.72 (1.35–2.20)	2E-04
	MAF	857 (29.8%)	485 (32.4%)	372 (27.1%)	834 (30.0%)	323 (24.0%)	511 (35.0%)	2.51E-4	0.002			2.27E-9		
	*P*_HWE_	0.9	0.13	0.07	0.28	0.14	0.46							
*SYTL3*	A/A	790 (55.0%)	386 (51.5%)	404 (58.8%)	854 (61.3%)	409 (62%)	445 (60.7%)							
rs2129209 A > C	A/C + C/C	646 (45.0%)	363 (48.5%)	283 (41.2%)	539 (38.7%)	251 (38%)	288 (39.3%)	0.001	0.006	0.77 (0.61–0.97)	0.025	0.63	0.99 (0.78–1.27)	0.95
	MAF	753 (26.2%)	421 (28.1%)	332 (24.2%)	606 (22.0%)	279 (21.0%)	327 (22.0%)	8.42E-5	0.016			0.46		
	*P*_HWE_	0.27	0.93	0.08	0.87	0.82	0.59							
*SYTL3*	A/A	1,107 (77.1%)	563 (75.2%)	544 (79.2%)	1,074 (77.1%)	507 (76.8%)	567 (77.3%)							
rs9456350 A > G	A/G + G/G	329 (22.9%)	186 (24.8%)	143 (20.8%)	319 (22.9%)	153 (23.2%)	166 (22.6%)	0.99	0.07	0.85 (0.64–1.11)	0.23	0.81	0.92 (0.69–1.21)	0.54
	MAF	349 (12.2%)	195 (13.0%)	154 (11.4%)	340 (12.0%)	162 (12.0%)	178 (12.0%)	0.95	0.14			0.92		
	*P*_HWE_	0.9	0.33	0.34	0.90	0.86	0.61							
*SLC22A3*	C/C	515 (35.9%)	357 (47.7%)	158 (23%)	412 (29.6%)	247 (37.4%)	165 (22.5%)							
rs446809 C > A	C/A + A/A	921 (64.1%)	392 (52.3%)	529 (77%)	981 (70.4%)	413 (62.6%)	568 (77.5%)	0.000368	2.13E-22	1.90 (1.53–2.37)	<0.0001	1.13E-9	1.91 (1.44–2.52)	<0.0001
	MAF	1,162 (40.5%)	470 (31.4%)	692 (50.4%)	1,275 (46.0%)	531 (40.0%)	744 (51.0%)	1.82E-18	3.89E-25			2.60E-8		
	*P*_HWE_	0.23	0.13	0.09	0.83	0.08	0.07							
*SLC22A3*	A/A	785 (54.7%)	390 (52.1%)	395 (57.5%)	756 (54.3%)	323 (48.9%)	433 (59.1%)							
rs539298 A > G	A/G + G/G	651 (45.3%)	359 (47.9%)	292 (42.5%)	637 (45.7%)	337 (51.1%)	300 (40.9%)	0.763	0.039	0.73 (0.58–0.92)	0.008	5.11E-8	0.55 (0.42–0.71)	2.0E-04
	MAF	755 (26.3%)	411 (27.4%)	344 (25.0%)	748 (27.0%)	403 (31.0%)	345 (24.0%)	0.633	0.144			3.12E-5		
	*P*_HWE_	0.54	0.46	0.07	0.15	0.41	0.36							

**SYTL3*, the synaptotagmin-like 3 gene; *SLC22A3*, the solute carrier family 22 member 3 gene; *HLP*, hyperlipidemia; *HWE*, Hardy–Weinberg equilibrium; *MAF*, minor allele frequency. *^∗^P*-value defined as chi-square test probability. *^∗∗^P*-value defined as logistic test probability.*

### The Relationship Between the Dominant Model of Six SNPs and the Risk of Hyperlipidemia

As shown in Table 2, the dominant model of rs446809 SNP increased HLP-related morbidity risk in both ethnic groups, whereas the dominant model of rs539298 SNP reduced HLP-associated morbidity in both the Han and Maonan groups. The dominant models of rs6455600 and rs2129209 SNPs decreased morbidity of HLP in the Han ethnicity, whereas the dominant model of rs6455600 SNP raised HLP-related morbidity in the Maonan ethnicity.

### The Relationship Between Genotypes of Six SNPs and Serum Lipid Levels

**FIGURE 2:**
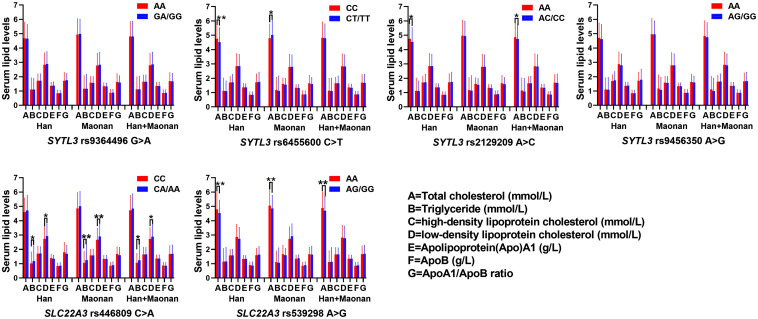
The association between the genotypes of *SYTL3* and *SLC22A3* single-nucleotide polymorphisms (SNPs) and serum lipid levels in the Han and Maonan ethnic groups. *P < 0.0083, **P < 0.001 [*P* < 0.0083 was considered statistically significant after Bonferroni correction; cigarette smoking, gender, blood pressure, body mass index (BMI), alcohol consumption, blood glucose, and age were adjusted for the statistical analyses].

As shown in Figure 2, several SNPs were associated with TC (rs6455600, rs2129209, and rs539298), TG (rs446809), and LDL-C (rs446809) levels in the Han and with TC (rs6455600 and rs539298), TG (rs446809), and LDL-C (rs446809) in the Maonan ethnic groups (*P* < 0.008–0.001 and *P* < 0.008, respectively, had statistical significance after Bonferroni correction).

### The Relationship Between the Haplotypes and Serum Lipid Levels

**FIGURE 3:**
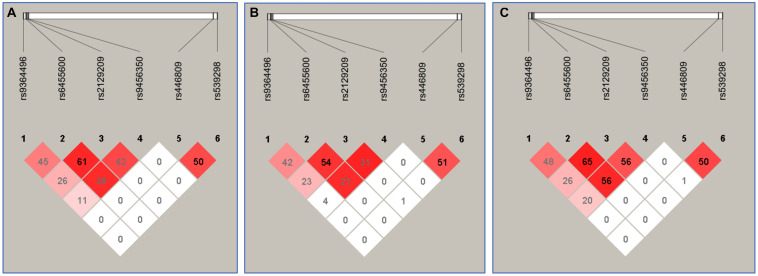
The linkage disequilibrium (LD) represents pair-wise *r*^2^ × 100 in Han + Maonan **(A)**, Han **(B)**, and Maonan **(C)** groups.

**FIGURE 4:**
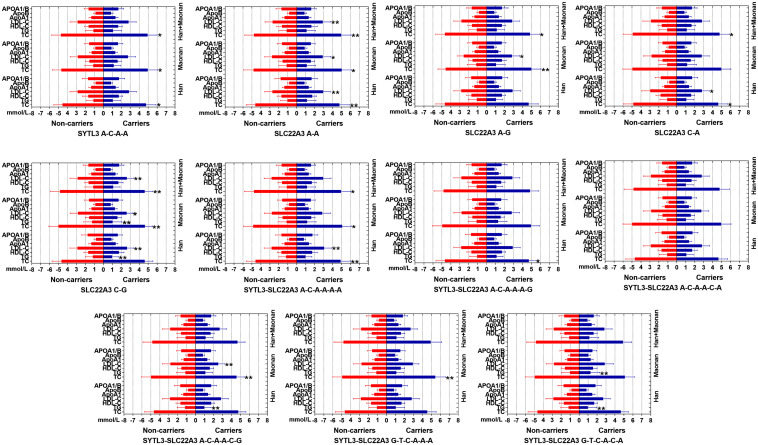
Lipid parameters according to the haplotypes of the Han and Maonan ethnic groups. *TC*, total cholesterol; *TG*, triglyceride; *HDL-C*, high-density lipoprotein cholesterol; *LDL-C*, low-density lipoprotein cholesterol; *Apo*, apolipoprotein. *P < 0.0045, **P < 0.001. (*P* < 0.0045 was considered statistically significant after Bonferroni correction; cigarette smoking, gender, blood pressure, BMI, alcohol consumption, blood glucose, and age were adjusted for the statistical analyses).

Figure 3 suggests the presence of significant pair wise LD among selected loci across both ethnic groups. Figure 4 indicates the associations of 11 haplotypes with blood lipid levels. Several haplotypes were correlated with TC (*SYLT3* A-C-A-A; *SLC22A3* A-A and C-A; and *SYTL3–SLC22A3* A-C-A-A-A-A and A-C-A-A-A-G), LDL-C (*SLC22A3* A-A, C-A, and C-G and *SYTL3–SLC22A3* A-C-A-A-A-A), and TG (*SLC22A3* C-G and *SYTL3–SLC22A3* A-C-A-A-C-G and G-T-C-A-C-A) in the Han and with TC (*SYLT3* A-C-A-A; *SLC22A3* A-A, A-G, and C-G; and *SYTL3–SLC22A3* A-C-A-A-A-A, A-C-A-A-C-G, and G-T-C-A-A-A), LDL-C (*SLC22A3* A-A, A-G, and C-G and *SYTL3–SLC22A3* A-C-A-A-C-G), and TG (*SLC22A3* C-G and *SYTL3–SLC22A3* G-T-C-A-A-A) in the Maonan ethnic groups (*P* < 0.0045–0.001 and *P* < 0.0045, respectively, had statistical significance after Bonferroni correction).

### The Relationship Between the Haplotypes and the Risk of Hyperlipidemia

As illustrated in Tables 3, 4, the dominant haplotype and G × G interaction haplotype were the *SYTL3* A-C-A-A (>55% of the samples) and *SYTL3–SLC22A3* A-C-A-A-C-A (>29% of the samples). The haplotypes of the *SYTL3* A-C-A-A; *SLC22A3* A-A and A-G; and *SYTL3–SLC22A3* A-C-A-A-A-A raised HLP-linked morbidity in both the Maonan and Han ethnicities, whereas the haplotypes of the *SLC22A3* C-G and *SYTL3–SLC22A3* A-C-A-A-C-G decreased HLP-related morbidity in both ethnic groups. In the Han group, the haplotypes of the *SLC22A3* C-A and *SYTL3–SLC22A3* A-C-A-A-C-A and G-T-C-A-C-A had a protective effect, while the haplotype of *SYTL3–SLC22A3* A-C-A-A-A-G increased HLP-related morbidity. In the Maonan group, the haplotypes of *SYTL3–SLC22A3* G-T-C-A-A-A and G-T-C-A-C-A raised HLP-related morbidity (*P* < 0.05–0.001).

**TABLE 3:** Association between the haplotypes among four SNPs of the *SYTL3* and two SNPs of the *SLC22A3* and hyperlipidemia in the Han and Maonan group [*n* (frequency)].

No.	Haplotypes	Han				Maonan			
			
		Normal	HLP	OR (95% CI)	*P-*value	Normal	HLP	OR (95% CI)	*P-*value
S1	A-C-A-A	865.99 (0.578)	874.69 (0.637)	1.257 (1.078–1.467)	0.003547	881.56 (0.668)	1,071.52 (0.728)	1.326 (1.082–1.597)	0.003203
S2	A-T-C-A	85.23 (0.057)	68.66 (0.050)	0.862 (0.621–1.195)	0.372051	34.04 (0.026)	45.23 (0.031)	1.189 (0.757–1.868)	0.451714
S3	A-T-C-G	122.23 (0.082)	93.79 (0.068)	0.815 (0.616–1.078)	0.150679	103.05 (0.079)	111.91 (0.076)	0.964 (0.729–1.275)	0.795912
S4	G-C-A-A	121.44 (0.081)	118.01 (0.086)	1.053 (0.808–1.372)	0.704067	95.90 (0.073)	117.58 (0.080)	1.100 (0.830–1.458)	0.506725
S5	G-T-C-A	133.37 (0.089)	110.53 (0.080)	0.884 (0.679–1.151)	0.360768	110.14 (0.084)	135.07 (0.092)	1.102 (0.846–1.435)	0.472837
Sa	A-A	271.13 (0.181)	336.91 (0.245)	1.470 (1.228–1.760)	2.63E-005	330.86 (0.253)	479.52 (0.327)	1.433 (1.214–1.691)	2.07E-005
Sb	A-G	198.87 (0.133)	226.09 (0.165)	1.287 (1.047–1.582)	0.016568	194.14 (0.149)	264.48 (0.180)	1.261 (1.030–1.544)	0.024729
Sc	C-A	815.87 (0.545)	693.09 (0.504)	0.851 (0.735–0.986)	0.031137	581.14 (0.445)	641.48 (0.438)	0.970 (0.835–1.128)	0.695241
Sd	C-G	117.91 (0.086)	212.13 (0.142)	0.569 (0.448–0.722)	2.90E-006	199.86 (0.153)	80.52 (0.055)	0.322 (0.245–0.422)	1.55E-015

*The haplotypes of S1–S5 are combined with *SYTL3* rs9364496-rs6455600-rs2129209-rs9456350 and Sa–Sb are combined with *SLC22A3* rs446809-rs539298. HLP, hyperlipidemia; *SYTL3*, the synaptotagmin-like 3 gene; *SLC22A3*, the solute carrier family 22 member 3 gene. Rare Hap (frequency < 1%) in both populations has been dropped. *P* was obtained by unconditional logistic regression analysis.*

**TABLE 4:** Association between the G × G interaction haplotypes among six SNPs of the *SYTL3*–*SLC22A3* cluster and hyperlipidemia in the Han and Maonan groups [*n* (frequency)].

No.	Haplotypes	Han				Maonan			
			
	A-B-C-D-E-F	Normal	HLP	OR (95% CI)	*P-*value	Normal	HLP	OR (95% CI)	*P-*value
H1	A-C-A-A-A-A	139.72 (0.093)	222.41 (0.162)	1.681 (1.336–2.114)	8.03E-006	211.50 (0.162)	325.61 (0.222)	1.842 (1.566–2.079)	1.64E-005
H2	A-C-A-A-A-G	125.72 (0.084)	173.59 (0.126)	1.406 (1.100–1.798)	0.006314	129.77 (0.099)	145.38 (0.099)	1.007 (0.782–1.296)	0.959564
H3	A-C-A-A-C-A	490.93 (0.328)	404.18 (0.294)	0.701 (0.593–0.828)	3.12E-005	416.39 (0.315)	433.06 (0.295)	0.907 (0.764–1.076)	0.261315
H4	A-C-A-A-C-G	110.59 (0.074)	74.49 (0.054)	0.634 (0.467–0.861)	0.003271	121.26 (0.093)	55.67 (0.038)	0.383 (0.275–0.532)	3.97E-009
H5	A-T-C-A-C-A	57.69 (0.039)	50.43 (0.037)	0.846 (0.575–1.246)	0.396886	50.43 (0.037)	62.72 (0.049)	1.270 (0.853–1.889)	0.238432
H6	A-T-C-G-C-A	65.07 (0.043)	59.91 (0.044)	0.893 (0.622–1.280)	0.536630	46.22 (0.035)	66.48 (0.045)	1.309 (0.891–1.923)	0.169696
H7	G-C-A-A-C-A	51.66 (0.034)	75.35 (0.035)	1.051 (0.809–1.288)	0.643759	44.83 (0.034)	40.40 (0.028)	0.802 (0.520–1.236)	0.316518
H8	G-T-C-A-A-A	44.07 (0.033)	39.12 (0.027)	0.809 (0.521–1.258)	0.346217	25.76 (0.017)	53.51 (0.039)	2.074 (1.287–3.343)	0.002240
H9	G-T-C-A-C-A	48.26 (0.032)	21.36 (0.016)	0.421 (0.251–0.706)	0.000732	38.56 (0.029)	64.52 (0.044)	1.537 (1.022–2.310)	0.037629

*A, rs9364496 G > A; B, rs6455600 C > T; C, rs2129209 A > C; D, rs9456350 A > G; E, rs446809 C > A; F, rs539298A > G. HLP, hyperlipidemia; SYTL3, the synaptotagmin-like 3 gene; SLC22A3, the solute carrier family 22 member 3 gene. Rare Hap (frequency < 1%) in both populations has been dropped. *P* was obtained by unconditional logistic regression analysis.*

### Several Gene–Gene/Environment Interactions on the Risk of HLP Based on GMDR

As presented in Table 5, several significant three-loci models comprising of SNP–SNP (rs9364496, rs6455600, and rs446809, possessing a testing accuracy of 56.71% and a CVC of 9 of 10), SNP–environment (rs446809 and rs539298 and BMI > 24 kg/m^2^, possessing a testing accuracy of 70.88% and a CVC of 10 of 10), haplotype–haplotype (*SYTL3* A-C-A-A and *SLC22A3* A-G and C-G, possessing a testing accuracy of 60.65% and a CVC of 10 of 10), haplotype–environment (*SLC22A3* A-G and C-G and BMI > 24 kg/m^2^, possessing a testing accuracy of 70.42% and a CVC of 10 of 10), gene–gene (*SYTL3–SLC22A3* A-C-A-A-A-A, A-C-A-A-A-G, and G-T-C-A-C-A, possessing a testing accuracy of 58.86% and a CVC of 10 of 10), and gene–environment (*SYTL3–SLC22A3* A-C-A-A-A-G and G-T-C-A-C-A and BMI > 24 kg/m^2^, possessing a testing accuracy of 58.45% and a CVC of 10 of 10) interactions were detected in the Han ethnicity (*P* < 0.001, respectively). Additionally, several different significant three-loci models comprising of SNP–SNP (rs6455600, rs446809, and rs539298, possessing a testing accuracy of 64.63% and a CVC of 10 of 10), SNP–environment (rs446809 and rs539298 and BMI > 24 kg/m^2^, possessing a testing accuracy of 70.83% and a CVC of 10 of 10), haplotype–haplotype (*SYTL3* A-C-A-A and *SLC22A3* A-A and A-G, possessing a testing accuracy of 58.87% and a CVC of 10 of 10) haplotype–environment (*SLC22A3* A-A and A-G and BMI > 24 kg/m^2^, possessing a testing accuracy of 58.85% and a CVC of 10 of 10), gene–gene (*SYTL3–SLC22A3* A-C-A-A-C-A, A-C-A-A-C-G, and A-T-C-G-C-A, possessing a testing accuracy of 57.18% and a CVC of 10 of 10), and gene–environment (*SYTL3–SLC22A3* A-C-A-A-C-A and A-C-A-A-C-G and BMI > 24 kg/m^2^, possessing a testing accuracy of 57.88% and a CVC of 8 of 10) interactions were also observed in the Maonan ethnicity (*P* < 0.0041–0.001, respectively).

**TABLE 5:** Different interactions among the SNPs, their haplotypes, and genetic and environmental factors detected by GMDR analyses.

Locus no.	Best combination	Training Bal.Acc	Testing Bal.Acc	Cross-validation consistency	*P*	^#^*P*
**Han**						
SNP–SNP interactions						
2	rs9364496 rs6455600	0.5849	0.5659	8/10	0.0010	<0.001
3	rs9364496 rs6455600 rs446809	0.6021	0.5671	9/10	0.0010	<0.001
SNP–environment interactions						
2	rs539298 BMI > 24 kg/m^2^	0.6946	0.6881	7/10	0.0010	<0.001
3	rs446809 rs539298 BMI > 24 kg/m^2^	0.7138	0.7088	10/10	0.0010	<0.001
Haplotype–haplotype interactions						
2	Sb Sd	0.6061	0.6065	10/10	0.0010	<0.001
3	S1 Sb Sd	0.6348	0.6174	10/10	0.0010	<0.001
Haplotype–environment interactions						
2	Sd BMI > 24 kg/m^2^	0.6816	0.6817	10/10	0.0010	<0.001
3	Sb Sd BMI > 24 kg/m^2^	0.7038	0.7042	10/10	0.0010	<0.001
Gene–gene interactions						
2	H2 H9	0.5711	0.5598	9/10	0.0010	<0.001
3	H1 H2 H9	0.5820	0.5886	10/10	0.0010	<0.001
Gene–environment interactions						
2	H2 H9	0.5681	0.5732	9/10	0.0010	<0.001
3	H2 H9 BMI > 24 kg/m^2^	0.5726	0.5845	10/10	0.0010	<0.001
**Maonan**						
SNP–SNP interactions						
2	rs446809 rs539298	0.6014	0.5628	9/10	0.0107	0.0041
3	rs6455600 rs446809 rs539298	0.6475	0.6463	10/10	0.0010	<0.001
SNP–environment interactions						
2	rs539298 BMI > 24 kg/m^2^	0.6623	0.6423	7/10	0.0010	<0.001
3	rs446809 rs539298 BMI > 24 kg/m^2^	0.7124	0.7083	10/10	0.0010	<0.001
Haplotype–haplotype interactions						
2	Sa Sb	0.5794	0.5761	10/10	0.0010	<0.001
3	S1 Sa Sb	0.5875	0.5887	10/10	0.0010	<0.001
Haplotype–environment interactions						
2	Sa Sb	0.5794	0.5761	8/10	0.0010	<0.001
3	Sa Sb BMI > 24 kg/m^2^	0.5883	0.5885	10/10	0.0010	<0.001
Gene–gene interactions						
2	H3 H4	0.5666	0.5672	10/10	0.0010	<0.001
3	H3 H4 H6	0.5716	0.5718	10/10	0.0010	<0.001
Gene–environment interactions						
2	H4 BMI > 24 kg/m^2^	0.5864	0.5868	10/10	0.0010	<0.001
3	H3 H4 BMI > 24 kg/m^2^	0.5957	0.5788	8/10	0.0107	0.0041

*S1 = *SYTL3*A-C-A-A haplotypes; Sa = *SLC22A3*A-A haplotypes; Sb = *SLC22A3*A-G haplotypes; Sd = *SLC22A3*C-G haplotypes; H1 = *SYTL3–SLC22A3* A-C-A-A-A-A; H2 = *SYTL3–SLC22A3* A-C-A-A-A-G; H3 = *SYTL3–SLC22A3* A-C-A-A-C-A; H4 = *SYTL3–SLC22A3* A-C-A-A-C-G; H6 = *SYTL3–SLC22A3* A-T-C-G-C-A; H9 = *SYTL3–SLC22A3* G-T-C-A-C-A. *P* was obtained by adjusting for height and weight. ^#^*P* was obtained by 1,000 permutation tests.*

### Visualization of Gene–Gene/Environment Interactions Based on MDR

**FIGURE 5:**
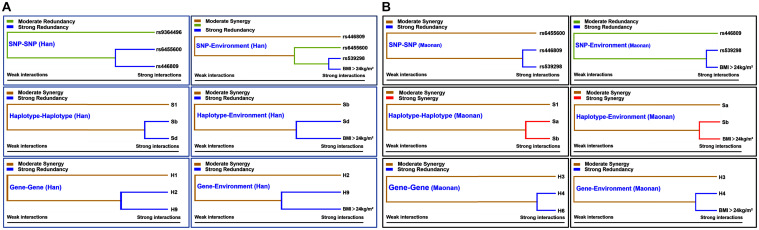
Different types of interaction dendrogram in the Han **(A)** and Maonan **(B)** groups. The strongly interacting elements appear close together at the leaves of the tree, and the weakly interacting elements appear distant from each other.

Figure 5 shows an entropy-based interaction dendrogram constructed based on MDR, which reveals the presence of a strong powerful redundancy interactions between the rs6455600 and rs446809 (SNP–SNP), rs539398 and BMI > 24 kg/m^2^ (SNP–environment), *SLC22A3* A-G and C-G (haplotype–haplotype), *SLC22A3* C-G and BMI > 24 kg/m^2^ (haplotype–environment), *SYTL3*–*SLC22A3* A-C-A-A-A-G and G-T-C-A-C-A (gene–gene), and *SYTL3*–*SLC22A3* G-T-C-A-C-A and BMI > 24 kg/m^2^ (gene–gene–environment) in the Han group. At the same time, there were strong powerful redundancy interactions between the rs446809 and rs539298 (SNP–SNP), rs539398 and BMI > 24 kg/m^2^ (SNP–environment), *SYTL3*–*SLC22A3* A-C-A-A-C-G and A-T-C-G-C-A (gene–gene), and *SYTL3*–*SLC22A3* A-C-A-A-C-G and BMI > 24 kg/m^2^ (gene–gene–environment) in the Maonan group. In addition, there were other strong powerful synergy interactions between *SLC22A3* A-A and A-G (haplotype–haplotype) and *SLC22A3* A-G and BMI > 24 kg/m^2^ (haplotype–environment) in the Maonan group.

### Unconditional Logistic Regression Analysis for the Interactions Between These Factors

As shown in Table 6, individuals bearing the rs6455600 CT/TT and rs446809 CA/AA genotypes increased the risk of HLP compared to those with the rs6455600 CC and rs446809 CC genotypes [adjusted odds ratio (OR) = 1.319, 95% confidence interval (CI) = 1.060–1.812, *P* = 0.008] in the Han group. The carriers of the rs539298 AG/GG genotypes and BMI > 24 kg/m^2^ (adjusted OR = 0.735, 95% CI = 0.482–0.924, *P* = 0.006), *SLC22A3* A-G and C-G (adjusted OR = 0.676, 95% CI = 0.523–0.874, *P* < 0.001), *SLC22A3* C-G and BMI > 24 kg/m^2^ (adjusted OR = 0.658, 95% CI = 0.408–0.861, *P* = 0.004), *SYTL3*–*SLC22A3* A-C-A-A-A-G and G-T-C-A-C-A (adjusted OR = 0.762, 95% CI = 0.612–0.915, *P* = 0.032), and *SYTL3*–*SLC22A3* G-T-C-A-C-A and BMI > 24 kg/m^2^ (adjusted OR = 0.649, 95% CI = 0.433–0.816, *P* = 0.011) reduced the risk HLP. On the other hand, carriers of the rs446809 CA/AA and rs539298 AG/GG (adjusted OR = 1.242, 95% CI = 1.018–1.522, *P* < 0.001), rs539298 AG/GG and BMI > 24 kg/m^2^ (adjusted OR = 1.263, 95% CI = 1.122–1.657, *P* < 0.001), *SLC22A3* A-A and A-G (adjusted OR = 1.838, 95% CI = 1.609–2.567, *P* < 0.001), *SLC22A3* A-G and BMI > 24 kg/m^2^ (adjusted OR = 2.360, 95% CI = 2.140–2.922, *P* < 0.001), and *SYTL3*–*SLC22A3* A-C-A-A-C-G and BMI > 24 kg/m^2^ (adjusted OR = 1.649, 95% CI = 1.359–1.901, *P* < 0.001) raised the risk of HLP in the Maonan group.

**TABLE 6:** Different types of interaction detected by logistic regression analyses.

Variable 1	Variable 2	OR (95% CI)	*P*-value
**Han**			
SNP–SNP interactions			
rs6455600	rs446809		
C/C	C/C	1	–
C/C	C/A + A/A	1.837 (1.346–2.507)	1.28E-4
C/T + T/T	C/C	0.683 (0.488–0.956)	0.026
C/T + T/T	C/A + –A/A	1.319 (1.060–1.812)	0.008
SNP–environment interactions			
rs539298	BMI > 24 kg/m^2^		
A/A	No	1	–
A/A	Yes	1.422 (1.107–1.846)	0.018
A/G + G/G	No	0.621 (0.436–0.806)	0.013
A/G + G/G	Yes	0.735 (0.482–0.924)	0.006
Haplotype–haplotype interactions			
Sb	Sd		
No-carriers	No-carriers	1	–
No-carriers	Carriers	0.455 (0.328–0.631)	2.38E-6
Carriers	No-carriers	1.016 (0.801–1.290)	0.894
Carriers	Carriers	0.676 (0.523–0.874)	4.58E-6
Haplotype–environment interactions			
Sd	BMI > 24 kg/m^2^		
No-carriers	No	1	–
No-carriers	Yes	1.357 (1.109–1.671)	0.036
Carriers	No	0.496 (0.347–0.671)	1.28E-4
Carriers	Yes	0.658 (0.408–0.861)	0.004
Gene–gene interactions			
H2	H9		
No-carriers	No-carriers	1	–
No-carriers	Carriers	0.683 (0.493–0.880)	0.016
Carriers	No-carriers	1.228 (1.036–1.619)	0.013
Carriers	Carriers	0.762 (0.612–0.915)	1.52E-5
Gene–environment interactions			
H9	BMI > 24 kg/m^2^		
No-carriers	No	1	–
No-carriers	Yes	1.226 (0.935–1.566)	0.543
Carriers	No	0.545 (0.393–0.776)	0.021
Carriers	Yes	0.649 (0.433–0.816)	0.011
**Maonan**			
SNP–SNP interactions			
rs446809	rs539298		
C/C	A/A	1	–
C/C	A/G + G/G	0.394 (0.348–0.581)	2.16E-7
C/A + A/A	A/A	1.386 (1.032–1.862)	0.030
C/A + A/A	A/G + G/G	1.242 (1.018–1.522)	1.56E-4
SNP–environment interactions			
rs539298	BMI > 24 kg/m^2^		
A/A	No	1	–
A/A	Yes	1.688 (1.220–2.336)	0.002
A/G + G/G	No	0.531 (0.404–0.698)	5.52E-6
A/G + G/G	Yes	1.263 (1.122–1.657)	0.023
Haplotype–haplotype interactions			
Sa	Sb		
No-carriers	No-carriers	1	–
No-carriers	Carriers	1.312 (1.072–1.613)	0.038
Carriers	No-carriers	1.426 (1.235–1.859)	0.013
Carriers	Carriers	1.838 (1.609–2.567)	3.22E-5
Haplotype–environment interactions			
Sb	BMI > 24 kg/m^2^		
No-carriers	No	1	–
No-carriers	Yes	1.793 (1.424–2.258)	6.33E-6
Carriers	No	1.204 (1.102–1.408)	0.033
Carriers	Yes	2.360 (2.140–2.922)	6.77E-6
Gene–gene interactions			
H4	H6		
No-carriers	No-carriers	1	–
No-carriers	Carriers	1.352 (0.794–1.819)	0.267
Carriers	No-carriers	0.488 (0.380–0.660)	1.81E-4
Carriers	Carriers	0.849 (0.659–1.401)	0.452
Gene–environment interactions			
H4	BMI > 24 kg/m^2^		
No-carriers	No	1	–
No-carriers	Yes	1.991 (1.572–2.520)	1.07E-8
Carriers	No	0.432 (0.328–0.816)	4.27E-4
Carriers	Yes	1.649 (1.359–1.901)	3.32E-5

**P* was obtained by adjusting for height and weight. The haplotypes of *SYTL3* were composed in the order of rs9364496, rs6455600, rs2129209, and rs9456350 SNPs. The haplotypes of *SLC22A3* were composed in the order of rs446809 and rs539298 SNPs. Gender, cigarette smoking, blood pressure, alcohol consumption, blood glucose, and age were adjusted for the statistical analysis.*

### The Relationship Among Lipid Parameters and Alleles/Genotypes and Several Haplotypes

The relationship between the genotypes and/or alleles of the six selected SNPs and blood lipid characteristics across both Han and Maonan ethnicities is shown in Supplementary Table 2. We noticed that the genotypes of rs6455600 and rs539298; and the haplotypes of *SYTL3* A-C-A-A, *SLC22A3* C-A and A-G, and *SYTL3–SLC22A3* A-C-A-A-A-A and A-C-A-A-A-G were associated with TC and the genotype of rs446809; and the haplotypes of *SLC22A3* C-G, and *SYTL3–SLC22A3* A-C-A-A-C-G and G-T-C-A-C-A were related to TG levels in the Han ethnicity. In the meantime, the allele of rs539298; the genotype of rs6455600; and the haplotypes of *SYTL3* A-C-A-A, SLC22A3 A-A, A-G and C-G; and *SYTL3–SLC22A3* A-C-A-A-A-A and A-C-A-A-C-G were associated with TC and the allele and genotype of rs6455600 and the haplotypes of *SLC22A3* A-G and C-G were related to TG in the Maonan ethnicity (*P* < 0.005–0.001).

### The Relationship Among Lipid Parameters and Environmental Exposures

The relationship between serum lipid parameters and numerous modifiable variants including waist circumference, gender, alcohol drinking, age, BMI, blood glucose, blood pressure, and cigarette smoking across both the Han and Maonan ethnic groups are shown in Supplementary Table 3 (*P* < 0.05–0.001).

### Relative Factors for Serum Lipid Parameters

**FIGURE 6:**
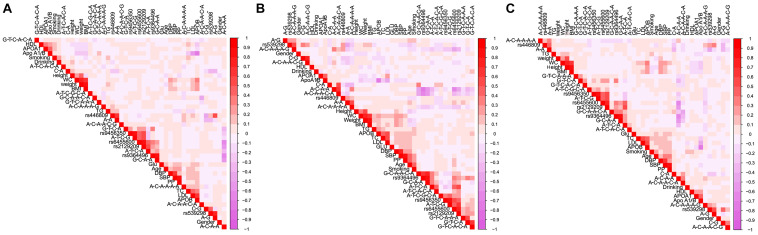
Correlations among environmental exposures and serum lipid variables, as well as the candidate loci and several haplotypes in Han + Maonan **(A)**, Han **(B)**, and Maonan **(C)** groups. *TC*, total cholesterol; *TG*, triglyceride; *HDL-C*, high-density lipoprotein cholesterol; *LDL-C*, low-density lipoprotein cholesterol; *ApoA1*, apolipoprotein A1; *ApoB*, apolipoprotein B; *ApoA1/B*, the ratio of apolipoprotein A1 to apolipoprotein B; BMI, body mass index; Glu, glucose; SBP, systolic blood pressure; DBP, diastolic blood pressure; WC, waist circumference.

Figure 6 is a Pearson correlation analysis that proves the relationship between haplotypes; integrative variants; the *SYTL3* rs9364496, rs6455600, rs2129209, and rs9456350 and *SLC22A3* rs446809 and rs539298 SNPs; and serum lipid profiles. Various modifiable factors comprising of alcohol consumption, cigarette smoking, age, gender, and several known cardiovascular risk factors including BMI, blood glucose, and blood pressure were also linked to blood lipid profiles in both the Maonan and Han ethnic groups.

## Discussion

The main findings of the current research included the following aspects: (1) *SYTL3*–*SLC22A3* SNPs and their haplotypes were linked to serum lipid profiles in the Han and Maonan ethnic groups, with variation in serum lipid profiles better predicted by haplotypes in comparison to any SNP alone, especially for TC and LDL-C. (2) The frequencies of six *SYTL3–SLC22A3* SNPs, their haplotypes, and gene–gene inter-locus interaction haplotypes in individuals of both Han and Maonan ethnicities provide complementary information to existing SNP-related data in the 1000 Genomes Project databases. (3) Several different interactions of the *SYTL3–SLC22A3* SNP–SNP/environment, gene–gene/environment, and haplotype–haplotype/environment on the risk of HLP were found in the Han and Maonan ethnic groups, and gene plays a dominant role in the interactions in the Han ethnic group, but environmental factor (BMI > 24 kg/m^2^) plays a dominant role in the interactions in the Maonan ethnic group. To the best of our knowledge, the associations between the SYTL3–SLC222A3 SNPs, their haplotypes, and gene–gene/environment interactions and blood lipid levels and the risk of hyperlipidemia in the Chinese populations have not been explored previously.

A series of studies has proved that HLP acts as a crucial risk factor for CAD, may be due to the combined effects of various factors including environmental exposures, genetic background, lifestyle, age, gender, and their interactions (Ruixing et al., 2007; Zhang et al., 2010). Maonan nationality, acting as one of the special minorities in Guangxi, maintained a higher cardiovascular risk compared with the local Han population enjoying the same social and natural environmental conditions. In a recent research, we noticed that the levels of ApoB, TC, LDL-C, and TG as well as the incidence of HLP in Maonan nationality were higher than those in Han nationality (Zheng et al., 2020). When we analyzed the frequencies of six *SYTL3–SLC22A3* SNPs in the Han and Maonan populations, we also noticed that the genotypic and allelic frequencies of the rs6455600, rs2129209, and rs446809 SNPs were suggestively distinctive between the Han and Maonan groups. Based on the data derived from the International 1,000 Genomes Project database^1^, the frequencies of rs9364496A allele and AA and AG genotypes were 24.1, 7.1, and 33.9%; rs6455600C allele and CC and CT genotypes were 40.3, 15.9, and 48.7%; rs2129209G allele and GG and GT genotypes were 24.6, 10.2, and 28.8%; rs9456350A allele and AA and AG genotypes were 39.8, 15.3, and 49.1%; rs446809A allele and AA and AC genotypes were 68.6, 44.2, and 48.7%; and rs539298A allele and AA and AG genotypes were 47.8, 23.0, and 49.6% in the European population, respectively. The frequencies of the above SNPs in the European population were significantly different from those in the Han or Maonan ethnic groups. It means that the frequencies of rare homozygous genotype or minor allele of six selected SNPs would be a shared racial/ethnic specificity.

Dietary habits, lifestyle, and marriage custom were significantly different between the Han and Maonan populations. The Maonan people still maintain their custom of intermarrying within the ethnic group, so their marriage customs are relatively conservative, and intermarrying with other ethnic groups is very rare. Therefore, perhaps, this is the main reason why the genetic characteristics and genotype frequencies of six selected SNPs were different between the Maonan and Han populations. Upon further analysis of the relationship between the genotypes of six SNPs and the occurrence of HLP, we noticed that the rs446809 CA/AA genotypes raised the risk of HLP while the rs539298 AG/GG genotypes decreased the risk of HLP in both Maonan and Han ethnicities. Interestingly, the rs2129209 AC/CC and rs6455600 CT/TT genotypes reduced the risk of HLP in the Han, while the rs6455600 CT/TT genotypes raised the risk of HLP in the Maonan ethnic groups. Thus, it can be seen that the difference in rs6455600 CT/TT frequencies may partly account for the different risk of HLP between the two ethnicities.

When the correlation between blood lipid profiles and the haplotypes of the six selected SNPs was analyzed, we discovered that there may be possible integrative haplotypes among them, which may be associated with TC (*SYLT3* A-C-A-A, *SLC22A3* A-A and C-A, and *SYTL3–SLC22A3* A-C-A-A-A-A and A-C-A-A-A-G), LDL-C (*SLC22A3* A-A, C-A, and C-G and *SYTL3–SLC22A3* A-C-A-A-A-A), and TG (*SLC22A3* C-G and *SYTL3–SLC22A3* A-C-A-A-C-G and G-T-C-A-C-A) in the Han and TC (*SYLT3* A-C-A-A; *SLC22A3* A-A, A-G, and C-G; and *SYTL3–SLC22A3* A-C-A-A-A-A, A-C-A-A-C-G, and G-T-C-A-A-A), LDL-C (*SLC22A3* A-A, A-G, and C-G and *SYTL3–SLC22A3* A-C-A-A-C-G), and TG (*SLC22A3* C-G and *SYTL3–SLC22A3* G-T-C-A-A-A) in the Maonan populations. Haplotype-based correlation analysis may have further impact on prediction of blood lipid levels in contrast to a single SNP analysis, especially with regard to TC and LDL-C.

Generalized multifactor dimensionality reduction analysis also detected several different interaction models among the gene–gene/environment, haplotype–haplotype/environment, and SNP–SNP/environment on HLP risk in the Han and Maonan ethnicities. We noticed that the participants from the Han group with the rs6455600 CT/TT and rs446809 CA/AA genotypes had increased HLP risk. The subjects with rs539298 AG/GG genotypes and BMI > 24 kg/m^2^, *SLC22A3* A-G and C-G, *SLC22A3* C-G and BMI > 24 kg/m^2^, *SYTL3–SLC22A3* A-C-A-A-A-G and G-T-C-A-C-A, and *SYTL3–SLC22A3* G-T-C-A-C-A and BMI > 24 kg/m^2^ in the Han group had decreased HLP risk. Additionally, the subjects with rs446809 CA/AA and rs539298 AG/GG genotypes, rs539298 AG/GG genotypes and BMI > 24 kg/m^2^, *SLC22A3* A-A and A-G, *SLC22A3* A-G and BMI > 24 kg/m^2^, and *SYTL3–SLC22A3* A-C-A-A-C-G and BMI > 24 kg/m^2^ in the Maonan group had increased HLP risk. These results indicated that genetic factors predominate in different SNP/haplotype/gene–environment interaction models in the Han group. However, BMI > 24 kg/m^2^ could reverse the functions of rs539298 SNP and *SYTL3–SLC22A3* A-C-A-A-C-G haplotype and also enhance the pathogenicity of *SLC22A3* A-G haplotype in the Maonan group. Thus, BMI > 24 kg/m^2^ acts as a crucial environmental factor to increase the incidence of HLP and is also a predominant characteristic across different SNP/haplotype/gene–environment interaction models in the Maonan group. Perhaps a reasonable explanation was that a genetic factor, combined with environmental factors, has been contributed to the development of HLP (Miao et al., 2018).

Several compelling studies have proven the strong relationship between blood lipid profiles and several environmental parameters such as exercise levels, hypertension, obesity, lifestyle habits, and diet (Mansfield et al., 1999; Valente et al., 2011; Joffe et al., 2013; Guo et al., 2017). Likewise, our investigation also produced a significant relationship between age, alcohol consumption, blood pressure, BMI, cigarette smoking, gender, and serum lipid profile in both the Han and Maonan groups, further lending credence to the gene–environment relationship involvement in clinical lipid parameters. Both ethnicities vary widely in terms of local customs and lifestyle habits. Besides rice, the Maonan diet is rich in carbohydrates such as potato, corn, sorghum, and wheat. Oil, spices, and salt are key features of their diet. Prolonged diets that contain high levels of saturated fat are known to result in atherosclerosis, obesity, high blood glucose levels, hypertension, and HLP (Ruixing et al., 2008a). Furthermore, it is known that long-term consumption of saturated fat imparts detrimental effects on lipid metabolism, especially TC and TG levels (Lottenberg et al., 2012). Atherosclerosis formation has also been shown to be influenced by different alcohol doses (Rimm et al., 1999). Moderate alcohol consumption may be protective against cardiovascular events, a phenomenon that has been attributed to elevated levels of ApoA1 and HDL-C (Ruixing et al., 2008b). Nevertheless, frequent heavy drinking leading to dyslipidemia, alcoholic fatty liver, and abnormal liver function is known to increase risk of CAD mortality (Pai et al., 2012). Several recent studies also have indicated that smoking (Maeda et al., 2003; Rao Ch and Subash, 2013) and excessive drinking (Ruixing et al., 2008b) were associated directly to HLP development and progression along with its complications. This investigation found that those of Maonan ethnicity were more likely to consume larger amounts of alcohol, while those with HLP were more likely to be smokers and alcohol consumers. Thus, the combined effects of lifestyle factors, various eating habits, and environmental aspects perhaps further alter the relationship of hereditary variations and serum lipid levels observed in the current research.

This research possesses several limitations. Firstly, this is a single-center study comprising of a small sample size in contrast to other GWASes. Secondly, we may have overlooked other potential lipid-related SNPs in addition to the six SNPs in the *SYTL3–SLC22A3* cluster. Thirdly, our study did not further explore the underlying mechanisms of actions of the identified SNPs in HLP development. Further *in vitro* and *in vivo* studies are necessary to strengthen the significance of our results.

## Conclusion

There are several potential associations between the *SYTL3–SLC22A3* SNPs, environment factors, and blood lipid spectrums in the Han and Maonan populations. Haplotypes may more accurately predict HLP in contrast to a single SNP analysis and explain more changes in lipid levels especially for TC and LDL-C levels. These SNPs and their haplotypes may account for some of the differences in serum lipid parameters between the Han and Maonan populations. Different interactions between SNP–SNP/environment, haplotype–haplotype/environment, and G × G/E interactions would produce different synergy or redundancy effects on the incidence of HLP in the Han and Maonan ethnic groups.

## Data Availability Statement

The raw data supporting the conclusions of this article will be made available by the authors, without undue reservation.

## Ethics Statement

The studies involving human participants were reviewed and approved by the Ethics Committee of The First Affiliated Hospital of Guangxi Medical University (No. Lunshen-2014 KY-Guoji-001, March 07, 2014). The patients/participants provided their written informed consent to participate in this study.

## Author Contributions

P-FZ conceived the study, participated in the design, performed the statistical analyses, and drafted the manuscript. R-XY and X-LC conceived the study, participated in the design, carried out the epidemiological survey, collected the samples, and helped to draft the manuscript. G-XD, Y-ZG, B-LW, and C-XL carried out the epidemiological survey and collected the samples. All authors read and approved the final manuscript.

## Conflict of Interest

The authors declare that the research was conducted in the absence of any commercial or financial relationships that could be construed as a potential conflict of interest.

## Footnotes

1 https://www.ncbi.nlm.nih.gov/variation/tools/1000genomes/
