# Supplementary material for: SYTL3–SLC22A3 Single-Nucleotide Polymorphisms and Gene–Gene/Environment Interactions on the Risk of Hyperlipidemia
Source: Front Genet. 2021 Jul 21;12:679027. doi: 10.3389/fgene.2021.679027 (PMC8334725; doi:10.3389/fgene.2021.679027)
Supplement: Supplementary file 1 [file Data_Sheet_1.docx]

**Supplementary Table 1.** The PCR primers for Next-Generation Sequencing

| SNP | Forwardprimer | Reverseprimer |
| --- | --- | --- |
| *SYTL3*rs9364496 | CCTGGCCTGTTGTATTTATAAGCA | CTCCTCCATGACCCTTCTAAGAGT |
| *SYTL3*rs6455600 | GTACCATATTCTCTGGAGGTTTTAGACAC | TGGATTGATAATGAATCTGCTGACACA |
| *SYTL3*rs2129209 | GCCTCAATAAATGTTTGTTGAGTGATT | CGCCTGTGGGATAGTGTGAAAAG |
| *SYTL3* rs9456350 | TTTTCTTGTGTCCTCCCATGGTAG | CCTGCGTGGAAAGCCAAATTTTT |
| *SLC22A3*rs446809 | AGGCATTAATCTCAAGGACACTCTT | GTGAAGCCATTAGACTTCACTTCTTAGT |
| *SLC22A3* rs539298 | ACTTTCTTCGAAGCCGAGTTGTAA | CCCTTGCATTTTGAGTAACCATTTGG |

*SYTL3*, synaptotagmin like 3; *SLC22A3,* solute carrier family 22 member 3.

**Supplementary Table 2.** Association of integrative *SYTL3* and *SLC22A3* mutations, haplotypes and G × G interaction haplotypes with lipid-related traits in the Han and Maonan populations

| **Lipid** | **SNP/ Haplotype** | **Affected**  **allele/**  **Other**  **allele** | **Affected**  **genotype/ Other**  **haplotype** | **Std.error** | **Beta** | ***t*** | ***P*** |
| --- | --- | --- | --- | --- | --- | --- | --- |
| **Han** |  |  |  |  |  |  |  |
| TC | H1 |  | Carriers/Non-carriers | 0.071 | 0.289 | 4.102 | 4.33E-5 |
|  | rs6455600 | CC/CT+TT |  | 0.062 | -0.257 | -4.154 | 3.45E-5 |
|  | Sc |  | Carriers/Non-carriers | 0.077 | -0.234 | -3.058 | 0.002 |
|  | Sa |  | Carriers/Non-carriers | 0.061 | 0.184 | 2.996 | 0.003 |
|  | S1 |  | Carriers/Non-carriers | 0.078 | 0.207 | 2.658 | 0.008 |
|  | H2 |  | Carriers/Non-carriers | 0.265 | -0.541 | -2.039 | 0.042 |
|  | rs539298 | AA/AG+GG |  | 0.058 | -0.114 | -1.974 | 0.048 |
| TG | Sd |  | Carriers/Non-carriers | 0.068 | 0.358 | 5.290 | 1.41E-7 |
|  | rs446809 | CC/CA+AA |  | 0.128 | -0.400 | -3.138 | 0.002 |
|  | H4 |  | Carriers/Non-carriers | 0.106 | 0.384 | 3.609 | 3.18E-4 |
|  | H9 |  | Carriers/Non-carriers | 0.154 | 0.353 | 2.288 | 0.022 |
| HDL-C | Sc |  | Carriers/Non-carriers | 0.034 | -0.110 | -3.185 | 0.001 |
|  | rs9456350 | A/G |  | 0.031 | 0.063 | 2.049 | 0.041 |
| LDL-C | H1 |  | Carriers/Non-carriers | 0.058 | 0.242 | 4.213 | 2.67E-5 |
|  | Sd |  | Carriers/Non-carriers | 0.060 | -0.253 | -4.233 | 2.45E-5 |
|  | Sa |  | Carriers/Non-carriers | 0.050 | 0.175 | 3.520 | 4.45E-4 |
|  | Sc |  | Carriers/Non-carriers | 0.058 | -0.167 | -2,861 | 0.004 |
|  | rs446809 | CC/CA+AA |  | 0.216 | -0.560 | -2.593 | 0.010 |
| APOA1 | rs446809 | C/A |  | 0.021 | 0.065 | 3.052 | 0.002 |
|  | H9 |  | Carriers/Non-carriers | 0.036 | 0.092 | 2.530 | 0.012 |
| APOB | Sd |  | Carriers/Non-carriers | 0.015 | -0.044 | -2.938 | 0.003 |
|  | H8 |  | Carriers/Non-carriers | 0.054 | -0.135 | -2.500 | 0.013 |
|  | rs9364496 | A/G |  | 0.025 | 0.048 | 1.967 | 0.049 |
| ApoA1/ApoB | H8 |  | Carriers/Non-carriers | 0.163 | 0.772 | 4.723 | 2.55E-6 |
|  | rs446809 | C/A |  | 0.035 | 0.137 | 3.889 | 1.05E-4 |
|  | H1 |  | Carriers/Non-carriers | 0.043 | -0.145 | -3.347 | 0.001 |
| **Maonan** |  |  |  |  |  |  |  |
| TC | Sd |  | Carriers/Non-carriers | 0.111 | -0.532 | -4.798 | 1.78E-6 |
|  | Sb |  | Carriers/Non-carriers | 0.134 | -0.599 | -4.477 | 8.19E-6 |
|  | rs6455600 | CC/CT+TT |  | 0.124 | 0.305 | 2.456 | 0.014 |
|  | rs539298 | A/G |  | 0.137 | 0.276 | 2.014 | 0.044 |
|  | S1 |  | Carriers/Non-carriers | 0.021 | 0.069 | 3.239 | 0.001 |
|  | Sa |  | Carriers/Non-carriers | 0.019 | 0.046 | 2.407 | 0.016 |
|  | H1 |  | Carriers/Non-carriers | 0.039 | -0.106 | -2.705 | 0.007 |
|  | H4 |  | Carriers/Non-carriers | 0.042 | -0.086 | -2.019 | 0.045 |
| TG | Sd |  | Carriers/Non-carriers | 0.091 | -0.359 | -3.958 | 7.95E-5 |
|  | H9 |  | Carriers/Non-carriers | 0.121 | -0.407 | -3.377 | 0.001 |
|  | rs446809 | CC/CA+AA |  | 0.114 | 0.540 | 4.720 | 2.59E-6 |
|  | rs6455600 | C/T |  | 0.115 | -0.454 | -3.955 | 8.05E-5 |
| HDL-C | S1 |  | Carriers/Non-carriers | 0.032 | -0.119 | -3.754 | 1.81E-4 |
|  | H6 |  | Carriers/Non-carriers | 0.058 | -0.16 | -2.775 | 0.006 |
|  | S2 |  | Carriers/Non-carriers | 0.031 | -0.094 | -3.036 | 0.002 |
| LDL-C | rs446809 | C/A |  | 0.093 | 0.567 | 6.163 | 9.34E-10 |
|  | Sd |  | Carriers/Non-carriers | 0.069 | -0.305 | -4.437 | 9.83E-6 |
|  | Sb |  | Carriers/Non-carriers | 0.103 | -0.381 | -3.685 | 2.38E-4 |
|  | Sa |  | Carriers/Non-carriers | 0.083 | -0.307 | -3.695 | 2.28E-4 |
|  | rs446809 | CC/CA+AA |  | 0.064 | -0.199 | -3.091 | 0.002 |
|  | H4 |  | Carriers/Non-carriers | 0.049 | -0.128 | -2.628 | 0.009 |
| ApoA1 | rs2129209 | A/C |  | 0.013 | -0.031 | -2.375 | 0.018 |
| ApoB | S2 |  | Carriers/Non-carriers | 0.020 | -0.083 | -4.064 | 5.11E-5 |
|  | H5 |  | Carriers/Non-carriers | 0.048 | 0.021 | 4.378 | 1.29E-5 |
|  | H6 |  | Carriers/Non-carriers | 0.016 | -0.066 | -4.028 | 5.92E-5 |
|  | rs6455600 | C/T |  | 0.025 | -0.105 | -4.134 | 3.78E-5 |
| ApoA1/ApoB | rs6455600 | C/T |  | 0.057 | 0.148 | 2.618 | 0.009 |
|  | rs9456350 | A/G |  | 0.115 | 0.266 | 2.311 | 0.021 |
|  | H9 |  | Carriers/Non-carriers | 0.088 | -0.212 | -2.400 | 0.017 |
|  | H1 |  | Carriers/Non-carriers | 0.059 | -0.287 | -4.856 | 1.33E-6 |
| **Han+Maonan** |  |  |  |  |  |  |  |
| TC | Sd |  | Carriers/Non-carriers | 0.066 | -0.176 | -2.662 | 0.008 |
|  | Sb |  | Carriers/Non-carriers | 0.074 | -0.596 | -8.101 | 8.04E-16 |
|  | Sc |  | Carriers/Non-carriers | 0.057 | -0.317 | -5.546 | 3.20E-8 |
|  | rs2129209 | A/C |  | 0.113 | -0.620 | -5.466 | 5.00E-8 |
|  | rs2129209 | AA/AC+CC |  | 0.114 | 0.530 | 4.668 | 3.19E-6 |
|  | rs446809 | C/A |  | 0.090 | 0.523 | 5.813 | 6.81E-9 |
|  | Sa |  | Carriers/Non-carriers | 0.076 | -0.531 | -6.996 | 3.29E-12 |
|  | rs539298 | A/G |  | 0.057 | -0.284 | -4.965 | 7.28E-7 |
|  | rs539298 | AA/AG+GG |  | 0.075 | -0.185 | -2.475 | 0.013 |
|  | H1 |  | Carriers/Non-carriers | 0.046 | 0.129 | 2.790 | 0.005 |
|  | S1 |  | Carriers/Non-carriers | 0.056 | 0.148 | 2.673 | 0.008 |
|  | S5 |  | Carriers/Non-carriers | 0.085 | 0.177 | 2.081 | 0.038 |
| TG | rs446809 | CC/CA+AA |  | 0.056 | -0.264 | -4.701 | 2.71E-6 |
|  | S2 |  | Carriers/Non-carriers | 0.070 | -0.403 | -5.738 | 1.06E-8 |
|  | rs446809 | C/A |  | 0.065 | 0.238 | 3.692 | 2.26E-4 |
|  | S3 |  | Carriers/Non-carriers | 0.070 | -0.176 | -2.523 | 0.012 |
| HDL-C | S3 |  | Carriers/Non-carriers | 0.027 | 0.149 | 5.402 | 7.14E-8 |
|  | H5 |  | Carriers/Non-carriers | 0.043 | -0.113 | -2.654 | 0.008 |
|  | rs446809 | C/A |  | 0.027 | -0.101 | -3.729 | 1.96E-4 |
| LDL-C | Sd |  | Carriers/Non-carriers | 0.050 | -0.178 | -3.561 | 3.76E-4 |
|  | H5 |  | Carriers/Non-carriers | 0.032 | -0.108 | -3.403 | 0.001 |
|  | rs446809 | CC/CA+AA |  | 0.035 | -0.095 | -2.712 | 0.007 |
|  | Sa |  | Carriers/Non-carriers | 0.042 | 0.105 | 2.527 | 0.012 |
| ApoA1 | rs446809 | C/A |  | 0.010 | -0.022 | -2.225 | 0.026 |
| ApoB | S5 |  | Carriers/Non-carriers | 0.018 | 0.042 | 2.326 | 0.020 |
|  | rs446809 | C/A |  | 0.014 | 0.065 | 4.781 | 1.83E-6 |
|  | rs539298 | A/G |  | 0.013 | -0.031 | -2.442 | 0.015 |
| ApoA1/ApoB | rs446809 | C/A |  | 0.034 | -0.184 | -5.440 | 5.77E-8 |
|  | rs6455600 | C/T |  | 0.062 | 0.162 | 2.634 | 0.008 |
|  | rs9456350 | A/G |  | 0.092 | 0.220 | 2.394 | 0.017 |

*HDL-C*, high density lipoprotein cholesterol; *LDL-C*, low density lipoprotein cholesterol; *Apo*, apolipoprotein.

**Supplementary Table 3**. Relationship between serum lipid parameters and relative environmental factors in the Han and Maonan populations

| **Lipid** | **Risk factor** | **B** | **Std.error** | **Beta** | ***t*** | ***P*** |
| --- | --- | --- | --- | --- | --- | --- |
| **Han** |  |  |  |  |  |  |
| TC | Cigarette smoking | 0.232 | 0.031 | 0.192 | 7.529 | 9.01E-14 |
|  | Diastolic blood pressure | 0.013 | 0.002 | 0.139 | 5.393 | 8.08E-8 |
|  | Age | 0.008 | 0.002 | 0.089 | 3.419 | 0.001 |
|  | Glucose | 0.016 | 0.034 | 0.055 | 2.136 | 0.033 |
| TG | Cigarette smoking | 0.253 | 0.034 | 0.199 | 7.498 | 1.13E-13 |
|  | Weight | 0.016 | 0.003 | 0.133 | 5.118 | 3.49E-7 |
|  | Systolic blood pressure | -0.007 | 0.001 | -0.119 | -4.461 | 3.79E-6 |
|  | Alcohol consumption | -0.092 | 0.045 | -0.054 | -2.044 | 0.041 |
| HDL-C | Cigarette smoking | 0.083 | 0.015 | 0.145 | 5.668 | 1.75E-8 |
|  | Glucose | -0.022 | 0.008 | -0.075 | -2.920 | 0.004 |
|  | Waist circumference | 0.002 | -0.010 | -0.163 | -6.217 | 6.64E-10 |
|  | Height | -0.007 | 0.002 | -0.108 | -4.139 | 3.69E-5 |
| LDL-C | Waist circumference | 0.007 | 0.004 | 0.070 | 1.941 | 0.032 |
|  | Age | 0.006 | 0.002 | 0.080 | 3.002 | 0.003 |
|  | Cigarette smoking | 0.014 | 0.026 | 0.142 | 5.383 | 8.57E-8 |
|  | BMI | 0.031 | 0.008 | 0.134 | 3.748 | 1.86E-4 |
|  | Alcohol consumption | -0.136 | 0.034 | -0.104 | -3.949 | 8.24E-5 |
|  | Systolic blood pressure | 0.003 | 0.001 | 0.080 | 2.952 | 0.003 |
|  | Glucose | 0.038 | 0.013 | 0.075 | 2.908 | 0.004 |
| ApoA1 | Weight | -0.004 | 0.001 | -0.150 | -5.815 | 7.49E-9 |
|  | Cigarette smoking | 0.041 | 0.008 | 0.139 | 5.372 | 9.09E-8 |
|  | Pulse pressure | 0.001 | 0.000 | 0.074 | 2.889 | 0.004 |
| ApoB | Waist circumference | 0.005 | 0.001 | 0.212 | 6.066 | 1.68E-9 |
|  | Systolic blood pressure | 0.001 | 0.000 | 0.116 | 4.567 | 5.37E-6 |
|  | Glucose | 0.012 | 0.003 | 0.097 | 3.865 | 1.16E-4 |
|  | BMI | 0.006 | 0.002 | 0.102 | 2.940 | 0.003 |
| ApoA1/ApoB | Waist circumference | -0.015 | 0.003 | -0.197 | -5.073 | 4.43E-7 |
|  | Weight | -0.006 | 0.003 | -0.092 | -2.735 | 0.018 |
|  | Cigarette smoking | 0.038 | 0.019 | 0.051 | 2.007 | 0.045 |
| **Maonan** |  |  |  |  |  |  |
| TC | Waist circumference | 0.018 | 0.003 | 0.161 | 5.658 | 1.86E-8 |
|  | Age | 0.007 | 0.002 | 0.093 | 3.323 | 0.001 |
|  | Diastolic blood pressure | 0.009 | 0.002 | 0.102 | 3.628 | 2.96E-4 |
|  | Height | -0.008 | 0.004 | -0.058 | -1.191 | 0.047 |
| TG | Waist circumference | 0.016 | 0.003 | 0.146 | 5.216 | 2.10E-7 |
|  | Cigarette smoking | 0.163 | 0.034 | 0.127 | 4.811 | 1.66E-6 |
|  | Alcohol consumption | -0.096 | 0.038 | -0.066 | -2.503 | 0.012 |
|  | Height | 0.009 | 0.004 | 0.063 | 2.224 | 0.026 |
| HDL-C | Waist circumference | -0.008 | 0.001 | -0.176 | -6.103 | 1.35E-9 |
|  | Diastolic blood pressure | 0.003 | 0.001 | 0.074 | 2.691 | 0.007 |
|  | Alcohol consumption | 0.040 | 0.016 | 0.069 | 2.593 | 0.010 |
|  | Height | 0.003 | 0.002 | 0.055 | 1.963 | 0.040 |
| LDL-C | Waist circumference | 0.017 | 0.003 | 0.180 | 6.130 | 1.21E-9 |
|  | Age | 0.123 | 0.028 | 0.132 | 4.399 | 1.19E-5 |
|  | Alcohol consumption | -0.133 | 0.035 | -0.114 | -3.793 | 1.57E-4 |
|  | Height | 0.040 | 0.015 | 0.079 | 2.732 | 0.006 |
|  | Diastolic blood pressure | 0.003 | 0.001 | 0.076 | 2.562 | 0.011 |
| ApoA1 | Waist circumference | -0.003 | 0.001 | -0.137 | -4.960 | 7.90E-7 |
|  | Alcohol consumption | -0.033 | 0.008 | -0.105 | -4.001 | 6.65E-5 |
|  | Systolic blood pressure | -0.001 | 0.000 | -0.124 | -2.953 | 0.003 |
|  | Cigarette smoking | -0.024 | 0.008 | -0.084 | -2.843 | 0.005 |
| ApoB | Waist circumference | 0.007 | 0.001 | 0.301 | 8.858 | 2.44E-18 |
|  | Cigarette smoking | 0.001 | 0.000 | 0.095 | 3.791 | 1.57E-4 |
|  | Alcohol consumption | 0.019 | 0.008 | 0.059 | 2.334 | 0.020 |
|  | Weight | -0.002 | 0.001 | -0.085 | -2.250 | 0.025 |
| ApoA1/ApoB | Waist circumference | -0.021 | 0.002 | -0.027 | -9.520 | 1.01E-20 |
|  | Cigarette smoking | 0.067 | 0.023 | 0.086 | 2.904 | 0.004 |
| **Han+Maonan** |  |  |  |  |  |  |
| TC | Diastolic blood pressure | 0.012 | 0.003 | 0.112 | 3.829 | 1.36E-4 |
|  | Cigarette smoking | 0.009 | 0.002 | 0.125 | 4.325 | 1.65E-5 |
|  | Waist circumference | 0.156 | 0.044 | 0.100 | 3.558 | 3.88E-4 |
|  | Age | 0.007 | 0.003 | 0.085 | 2.833 | 0.005 |
|  | Height | -0.009 | 0.003 | -0.060 | -3.056 | 0.002 |
| TG | Cigarette smoking | 0.130 | 0.026 | 1.113 | 4.994 | 6.79E-7 |
|  | Weight | 0.224 | 0.049 | 0.126 | 4.550 | 5.93E-6 |
|  | Age | -0.245 | 0.057 | -0.810 | -4.306 | 1.80E-5 |
|  | Alcohol consumption | -0.069 | 0.021 | -0.406 | -3.373 | 0.001 |
| HDL-C | Waist circumference | -0.008 | 0.001 | -0.159 | -8.548 | 4.55E-11 |
|  | Cigarette smoking | 0.112 | 0.022 | 0.142 | 5.063 | 4.78E-7 |
|  | Glucose | -0.016 | 0.001 | -0.108 | -3.699 | 2.27E-4 |
| LDL-C | Waist circumference | 0.017 | 0.003 | 0.189 | 6.259 | 5.39E-10 |
|  | Age | 0.007 | 0.002 | 0.131 | 4.390 | 1.24E-5 |
|  | Alcohol consumption | -0.148 | 0.032 | -0.126 | -4.542 | 6.13E-6 |
|  | Height | -0.012 | 0.004 | -0.103 | -3.345 | 0.001 |
|  | Systolic blood pressure | 0.004 | 0.002 | 0.074 | 3.645 | 2.72E-4 |
|  | Cigarette smoking | 0.063 | 0.019 | 0.062 | 3.296 | 0.001 |
| ApoA1 | Weight | -0.002 | 0.001 | -0.068 | -2.425 | 0.015 |
|  | Waist circumference | -0.004 | 0.001 | -0.162 | -5.672 | 1.77E-8 |
|  | Cigarette smoking | -0.030 | 0.009 | -0.091 | -3.170 | 0.002 |
| ApoB | Waist circumference | 0.008 | 0.001 | 0.335 | 8.457 | 7.94E-17 |
|  | Systolic blood pressure | 0.044 | 0.009 | 0.132 | 4.878 | 1.22E-6 |
|  | Cigarette smoking | 0.001 | 0.000 | 0.089 | 3.315 | 0.001 |
|  | Glucose | 0.038 | 0.013 | 0.081 | 2.967 | 0.003 |
| ApoA1/ApoB | Waist circumference | -0.015 | 0.002 | -0.274 | -9.801 | 7.3E-22 |
|  | Pulse pressure | -0.003 | 0.001 | -0.087 | -2.747 | 0.006 |

*HDL-C*, high density lipoprotein cholesterol; *LDL-C*, low density lipoprotein cholesterol; *Apo*, apolipoprotein.
